# How to improve automated external defibrillator placement for out-of-hospital cardiac arrests: A case study

**DOI:** 10.1371/journal.pone.0250591

**Published:** 2021-05-20

**Authors:** Dylan Aeby, Philippe Staeger, Fabrice Dami

**Affiliations:** 1 Faculty of Medicine, University of Lausanne, Lausanne, Switzerland; 2 Center for Primary Care and Public Health (Unisanté), University of Lausanne, Lausanne, Switzerland; 3 Emergency Department, Lausanne University Hospital, University of Lausanne, Lausanne, Switzerland; Fondazione IRCCS Policlinico San Matteo, ITALY

## Abstract

**Introduction:**

In out-of-hospital cardiac arrests (OHCAs), the use of an automatic external defibrillator (AED) by a bystander remains low, as AEDs may be misplaced with respect to the locations of OHCAs. As the distribution of historical OHCAs is potentially predictive of future OHCA locations, the purpose of this study is to assess AED positioning with regard to past locations of OHCAs, in order to improve the efficiency of public access defibrillation programs.

**Methods:**

This is a retrospective observational study from 2014 to 2018. The locations of historical OHCAs and AEDs were loaded into a geodata processing tool. Median distances between AEDs were collected, as well as the number and rates of OHCAs covered (distance of <100 meters from the nearest AED). Areas with high densities of uncovered OHCAs (hotspots) were identified in order to propose the placement of additional AEDs. Areas over-covered by AEDs (overlays) were also identified in order to propose the relocation of overlapping AEDs.

**Results:**

There were 2,971 OHCA, 79.3% of which occurred at home, and 633 AEDs included in the study. The global coverage rate was 7.5%. OHCAs occurring at home had a coverage rate of 4.5%. Forty hotspots were identified, requiring the same number of additional AEDs. The addition of these would increase the coverage from 7.5% to 17.6%. Regarding AED overlays, 17 AEDs were found to be relocatable without reducing the AED coverage of historical OHCAs.

**Discussion:**

This study confirms that geodata tools can assess AED locations and increase the efficiency of their placement. Historical hotspots and AED overlays should be considered, with the aim of efficiently relocating or adding AEDs. At-home OHCAs should become a priority target for future public access defibrillation programs as they represent the majority of OHCAs but have the lowest AED coverage rates.

## Introduction

Every year, 30.0 to 97.1 in 100,000 people suffer from an out-of-hospital cardiac arrest (OHCA), for which the survival rate varies from 3.1 to 20.4% according to different registries [1]. When defibrillation is provided within 3 to 5 minutes, it can significantly increase the survival rate [2], especially if coupled with effective chest compressions. However, in a large majority of cases, emergency medical services cannot reach the patient within this very short period of time [3]. Fortunately, defibrillation can be performed by bystanders if an automatic external defibrillator (AED) is available. Any OHCA taking place within a 100 m radius of an AED is considered to be "covered", as the device is reachable within 90 seconds by brisk walking, allowing defibrillation to be performed within 3–5 minutes [4–7], as recommended by the American Heart Association (AHA) [8]. Therefore, public authorities have launched public access defibrillation (PAD) programs, as recommended by the European Resuscitation Council Guidelines [2].

However, due to the cost of AEDs, it is only possible to place a limited number of them; therefore, their location must be methodical to ensure that they are as effective as possible. In addition to public AEDs, some AEDs are privately owned (by private communities or enterprises) and are placed based on different criteria (e.g., number of visitors, specific risks, marketing, to improve the company’s image), rather than following a well-conducted risk analysis [9].

Nevertheless, the use of an AED by a bystander remains low for a number of reasons. AEDs may be misplaced regarding location of OHCAs [4–6, 10–12], be inaccessible at the time of the OHCA [13–16] or not registered through the dispatch centre, or bystanders may have difficulty finding them because their locations are poorly indicated [17]. Finally, most OHCAs occur at home, often with no one else around, or with only a sole elderly witness, and these locations are less covered by AEDS [18].

Various studies have highlighted that OHCAs are distributed in clusters, which remain stable over the years [19–21]. Therefore, these locations may be suitable candidates for the placement of AEDs, as they are potentially predictive of future OHCAs.

The purpose of this study is to assess the positioning of AEDs with regard to historical locations of OHCAs, in order to improve the efficiency of PAD programs, and to describe the work method.

## Materials and methods

This is a retrospective observational study from January 1^st^ 2014 to December 31^st^ 2018 in the State of Vaud (Switzerland), a territory of 3,212.2 square kilometres with a population of 793,129 inhabitants. Its capital, Lausanne, has 148,000 inhabitants [22].

When the dispatch centre suspects an OHCA, including OHCAs occurring at home, an ambulance and an emergency physician with his own vehicle are dispatched. Simultaneously, lay responders are alerted via a web-application. If more than one lay responder is available and an AED is in the vicinity, one is directed to the victim and another toward the AED. Lay responders are not alerted for suspected traumatic OHCA. Police vehicles within the State are all equipped with AEDs and are registered within the State’s lay responder web-application. The dispatch centre has the locations of all public AEDs and most private ones, as the State strongly recommended that they are registered. For this study, all OHCAs for which an ambulance was dispatched were considered. Traumatic OHCAs, as well as OHCAs occurring on highways and in health care facilities (such as clinics, treatment and rehabilitation centres, medico-social establishments, medical practices, long term care and psychiatric hospitals) were excluded. OHCAs with an incomplete address were also excluded. The locations of the AEDs were obtained through the State’s dispatch centre. The following data were collected:

- AED location and type (public or private places), available 24/7 as of 31^st^ January 2019
- OHCA location (home or public places such as workplace, street, sports club) and cause (cardiac, respiratory, others non-cardiac causes) from January 1^st^ 2014 to December 31^st^ 2018

### Data processing

Base map and data are issue from OpenStreetMap and OpenStreetMap Foundation. The geolocalization analysis was performed on the Quantum Geographic Information System (QGIS, 3.2.3 Bonn), a free and open-source cross-platform desktop application that supports viewing, editing and analysis of geospatial data from the Geospatial Foundation Project. Addresses were converted into geographical locations with a longitude and latitude combination. This method has been validated in previous epidemiological studies [23–26].

### Median distances and coverage rate

The straight-line distance from each OHCA to the nearest AED (in meters) was computed by the software and then entered in a spreadsheet (Microsoft Excel, Microsoft Corp., Redmond, Washington, USA). Each OHCA was categorized into urban, intermediate or rural, according to the Swiss Federal Statistical Office [27]. OHCAs were also categorized in terms of the type of location (home vs public place). The median distances, interquartile range (IQR) and coverage rates (percentage of OHCAs covered in proportion to all OHCAs) were calculated for each category and type of location. In this study, according to the AHA recommendation, an OHCA where the nearest AED was within a 100 m radius was considered as “covered” [8].

### Hotspots and AED overlays

Areas with ≥5 historical OHCAs within a 100 m radius (public or private place) and without an AED were defined as “hotspots” by the study team. Areas with <5 historical OHCAs were arbitrarily not considered, as this may imply an unreasonable number of AEDs to add. Hotspots were identified with QGIS, by running a uniform density estimation algorithm, and then displayed on a map. These hotspots are recommended for AED placement as they are potentially predictive of future OHCAs. The impact on the coverage rates and median distances following the installation of AEDs in these hotspots was then determined.

Areas with a high density of AEDs (>1) within a 100 m radius were defined as AED “overlays”. They were identified using the same method. Public AEDs in such areas could be relocated elsewhere, without significant loss of coverage.

### Ethical approval

The project was submitted to the State’s Ethics committee. Due to the lack of clinical data from the patients, a formal request was deemed unnecessary as it is not a concern regarding the law on Human research

## Results

**Fig 1:**
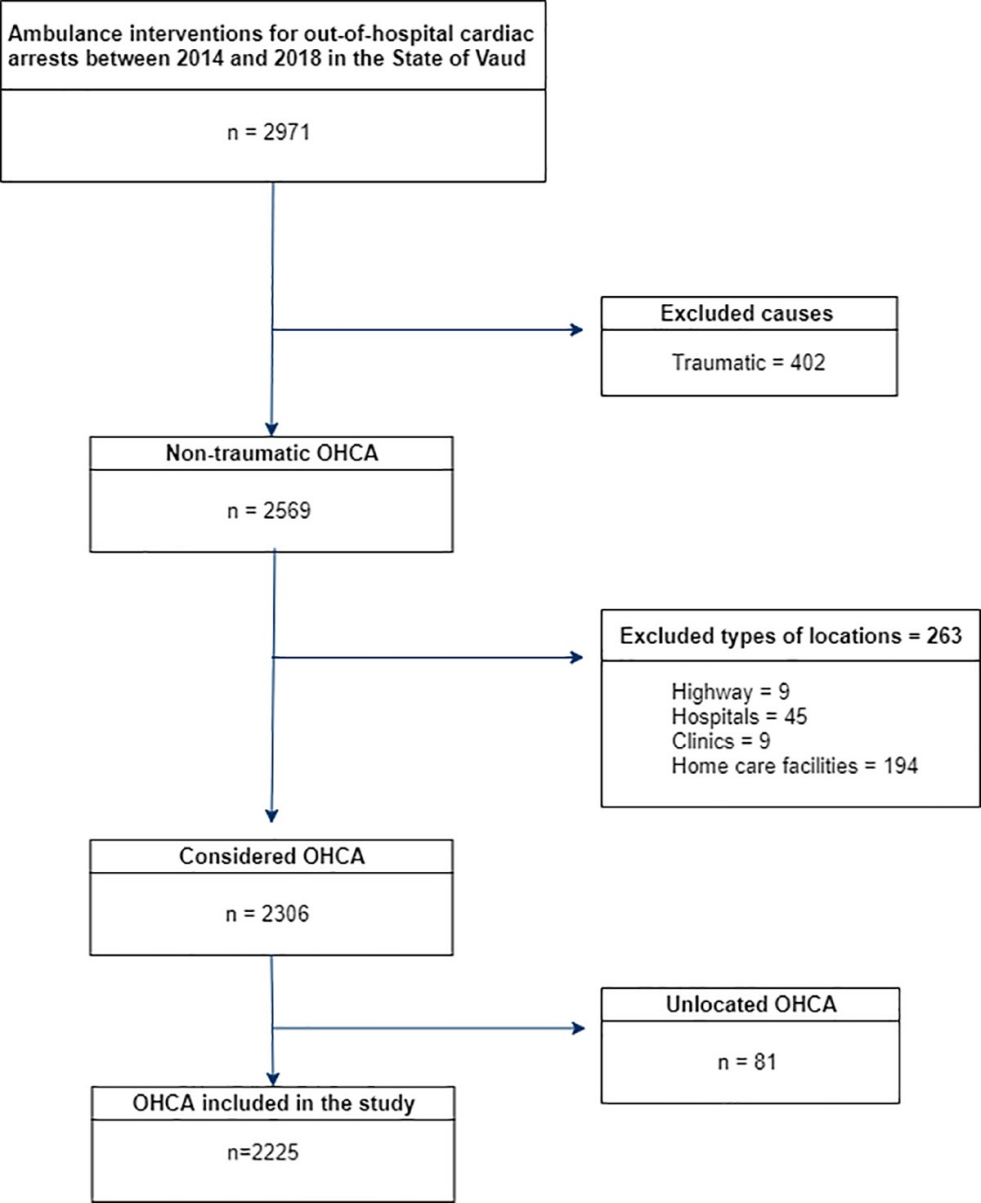
Flowchart.

During the study period, 2,971 OHCAs were eligible for inclusion, and 2,225 were finally included (Fig 1). There were 633 public and private registered AEDs available 24/7 on January 31^st^ 2019.

### Median distances and coverage rate

The median distance from each OHCA to the nearest AED is 410.9 m (IQR: 216–1004 m). This equates to median distances of 319.7 m (IQR: 184–538 m) in urban municipalities, 1874.7 m (IQR: 535–2846 m) in rural municipalities and 759.9 m (IQR: 351–1619 m) in intermediate municipalities (Table 1). The global AED coverage rate of historical OHCAs is 7.5% (167/2225), specifically 10.3% (144/1399) in urban municipalities, 2.9% (10/340) in rural municipalities and 2.7% (13/486) in intermediate municipalities (Table 1). In terms of the type of location, 1765 (79.3%) of OHCAs occurred at home. The median distance from the OHCAs that occurred at home to the nearest AED is 429.4 m (IQR: 243–1086 m), and the coverage rate is 4.5% (79/1765). The median distance to the nearest AED in out-of-home OHCAs (i.e., public areas, workplaces) is 303.1 m (IQR: 137–783 m), and the coverage rate is 19.1% (88/460), as shown in Table 2. Table 3 shows the results for Lausanne, the State’s capital.

**Table 1:** OHCA characteristics categorized by the type of municipality.

*Actual situation*	**OHCA**	**OHCA covered**	**Coverage rate**	**Median distance OHCA-nearest AED (meters)**	**Interquartile Range (IQR)**
**Urban municipalities**	1399	144	10.3%	319.7	(184;538)
**Intermediate municipalities**	486	13	2.7%	759.9	(351;1619)
**Rural municipalities**	340	10	2.9%	1874.7	(535;2846)
**Total**	2225	167	7.5%	410.9	(216;1004)
*Situations with AED on proposed location*	**OHCA**	**OHCA covered**	**Coverage rate**	**Median distance OHCA-nearest AED (meters)**	**Interquartile Range (IQR)**
**Urban municipalities**	1399	357 (+213)	25.5% (+15,2)	252.7 (-67)	(99 (-85); 481 (-57))
**Intermediate municipalities**	486	25 (+12)	5.1% (+2,4)	759.9 (+0)	(322 (-29); 1619 (+0))
**Rural municipalities**	340	10 (+0)	2.9% (+0)	1874.7 (+0)	(535 (+0); 2846 (+0))
**Total**	2225	392 (+225)	17.6% (+10,1)	368.6	(162 (-54); 984 (-20))

**Table 2:** OHCA characteristics categorized by location type.

*Actual situation*	**OHCA**	**OHCA covered**	**Coverage rate**	**Median distance OHCA-nearest AED (meters)**	**Interquartile Range (IQR)**
**Home**	1765	79	4.5%	429.4	(243;1086)
**Public**	460	88	19.1%	303.1	(137;783)
**Total**	2225	167	7.5%	410.9	(216;1004)
*Situations with AED on proposed location*	**OHCA**	**OHCA covered**	**Coverage rate**	**Median distance OHCA- nearest AED (meters)**	**Interquartile Range (IQR)**
**Home**	1765	264 (+185)	15.0% (+10.5)	387.5 (-41.9)	(185 (-58); 1086 (+0))
**Public**	460	128 (+40)	27.8% (+8.7)	254.2 (-48.9)	(97 (-40); 776 (-7))
**Total**	2225	392 (+225)	17.6% (+10.1)	368.6 (-42.3)	(162 (-54); 984 (-20))

**Table 3:** OHCA characteristics in Lausanne, the State’s capital.

*Actual situation*	**OHCA**	**OHCA covered**	**Coverage rate**	**Median distance OHCA-nearest AED (meters)**	**Interquartile Range (IQR)**
**Home**	318	24	7.5%	268.8	(179;385)
**Public**	119	36	30.3%	172.0	(93;292)
**Total**	437	60	13.7%	246.0	(152;372)
*Situations with AED on proposed location*	**OHCA**	**OHCA covered**	**Coverage rate**	**Median distance OHCA-nearest AED (meters)**	**Interquartile Range (IQR)**
**Home**	318	116 (+92)	36.5% (+29)	170.2 (- 98.6)	(89 (-90); 316 (-69))
**Public**	119	55 (+19)	46.2% (+15.9)	118.7 (-53.3)	(74 (-19); 205 (-87))
**Total**	437	171 (+111)	39.1% (+25,4)	159.5 (-86.5)	(87 (-65); 290 (-82))

### Hotspots and AED overlays

**Fig 2:**
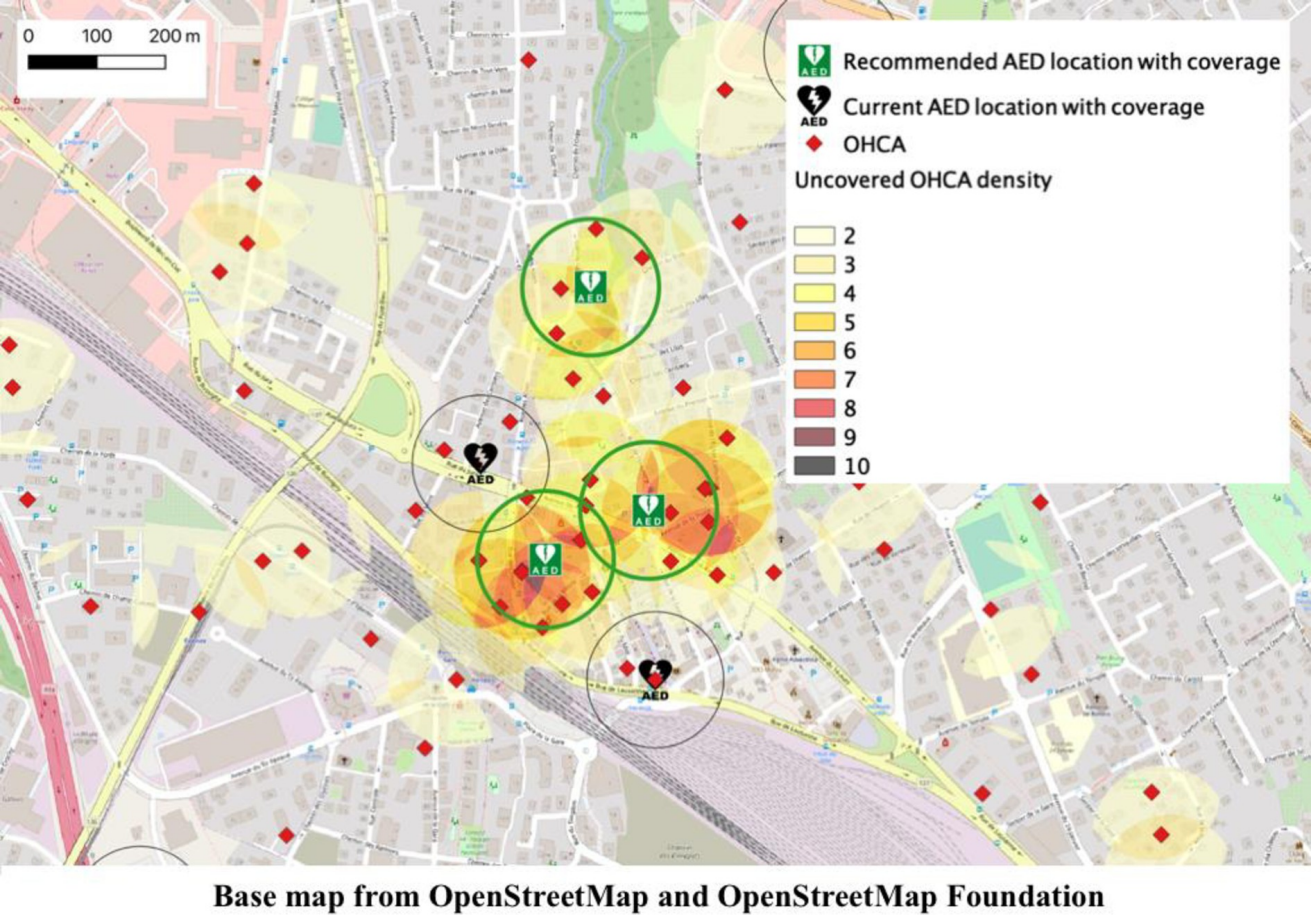
Map of the highest concentrations of uncovered OHCAs in the State. The density of uncovered OHCAs in an area is defined by the number of uncovered OHCAs located in a radius of 100 m.

There are 40 hotspots (≥ 5 OHCAs within a 100 m radius not covered by an AED): 38 in urban areas and 2 in intermediate locations. There are no hotspots in rural communities. If these hotspots were equipped with AEDs, 225 additional historical OHCAs (213 in urban areas and 12 in intermediate areas) would be covered. The coverage rate would then rise from 7.5% (167/2225) to 17.6% (392/2225). The impact of additional AEDs is shown in Tables 1–3. Fig 2 presents the area with the highest concentration of uncovered OHCAs and shows how adding three AEDs would improve the coverage. More hotspots and recommended AED placements can be found in the S1 File.

**Fig 3:**
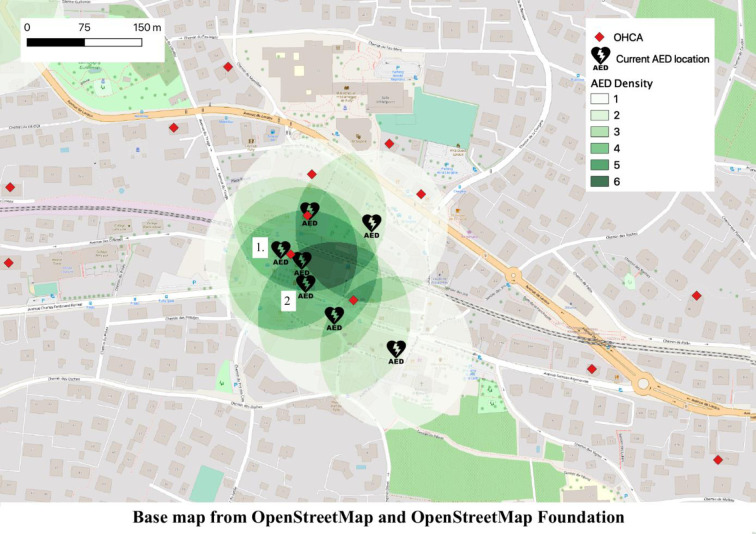
Map of the area with the highest AED density of the State of Vaud. The AED density of an area is defined by the number of AEDs within a 100 m radius.

Regarding AED overlays, 17 public AEDs could be relocated without reducing the AED coverage rate in the State. Fig 3 illustrates an example of severe overlap, where AEDs 1 and 2 could be removed. More suggestions to replace AEDs can be found in the S2 File.

S3 File provides a comparison of AED coverage rates from previous works and the present study.

## Discussion

This study proposes a method to evaluate the efficiency of the PAD coverage in an urban and rural territory. It confirms that it is possible to improve the spatial coverage rate of historical OHCAs by adding new AEDs and also points to a less common concept of relocating AEDs that are inefficiently placed (overlapping) to main hotspot areas. Adding an AED to each identified hotspot would increase the coverage rate and would require 40 AEDs for the whole State. Working on overlays and relocating overlapping AEDs, the example in our study shows that it would be possible to relocate 17 AEDs, thus sparing the number of new AEDs that would be required.

The study also confirms that the majority of OHCAs take place at home (79.3%) and that their coverage rate is poor (4.5%), four times lower than in public places (19.1%) [18].

The concept of improving the spatial or spatio-temporal AEDs coverage rate is not new [4, 6, 12, 28]; however, improving the efficiency of PADs by relocating AEDs is less known.

Tierney et al. developed a flexible location model for AEDs, which includes the relocation of existing AEDs to improve coverage [7]. The present work uses a less complex method but emphasizes the opportunity of saving costs while improving a public system. When thinking about adding new AEDs, every PAD program should consider the possibility of relocating existing AEDs to improve coverage.

Besides improving hotspots, OHCAs occurring at home deserve special attention, especially in high-density areas. They represent the majority of OHCAs, yet an AED is rarely available. Until now, PAD programs have mainly focused on public areas because the benefits on survival have been demonstrated [2]. In addition, some studies concluded that the conditions for public defibrillation in residential areas were less favourable for several reasons [2, 29–32], including a higher rate of non-shockable rhythms [33]. However, the context has changed with the emergence of lay responders who can be alerted by dispatch centres [34]. These programs show higher CPR rates [34–36], more frequent shockable rhythms [29, 37, 38] and faster defibrillations [37, 39–42]. Consequently, some authors are now calling for a paradigm shift from public, to all-access defibrillation [43]. This could be achieved by the expansion of AED networks in residential areas, accompanied by the development of systems of local lay responders [31, 40, 41, 44, 45].

### Limitations

There were certain limitations with this study. Firstly, distances were calculated as the crow flies; therefore, vertical distances (height in a building) were not taken into account. Secondly, existing and unregistered AEDs do not appear in this study, but they have a limited impact on population coverage because dispatchers are unable to guide an OHCA bystander or lay responder to these AEDs if they are not listed. Finally, the count of AEDs was stopped on 31^st^ January 2019, and more AEDs may have been registered since, thus increasing the coverage rate.

## Conclusion

Geodata tools can be used to increase the coverage of OHCAs with AEDs by detecting historical OHCA hotspots and identifying overlapping AEDs, which could be moved for better coverage efficiency. At-home OHCAs represent the majority of OHCAs, yet they are poorly covered. These should become a priority target for future PAD programs.

## Supporting information

S1 File Recommended locations for new AEDs. (DOCX)

S2 File Suggested public AEDs to be removed. (DOCX)

S3 File Comparison of AED coverage rates from previous works and the present study. (DOCX)

## Abbreviations

AED: automatic external defibrillator
EMS: emergency medical service
OHCA: out-of-hospital cardiac arrest
PAD: public access defibrillation
QGIS: Quantum geographic information system
