# Supplementary material for: How to improve automated external defibrillator placement for out-of-hospital cardiac arrests: A case study
Source: PLoS One. 2021 May 20;16(5):e0250591. doi: 10.1371/journal.pone.0250591 (PMC8136701; doi:10.1371/journal.pone.0250591)
Supplement: S1 File — (DOCX) [file pone.0250591.s001.docx]

# **S1 File. Recommended locations for new AEDs**

1. Bus station “Pierrefleur”, 1004 Lausanne
2. Bus station “Rapille”, 1004 Lausanne
3. Bergière’ Veterinarian's office, Av. Bergière 50, 1004 Lausanne
4. Rue des crêtes 26, 1018 Lausanne
5. Chemin Aimé-Steinlen 9, 1004 Lausanne
6. Avenue du Parc-de-la-Rouvraie 12, 1018 Lausanne
7. Chemin de la Joliette 8, 1006 Lausanne
8. Chemin Auguste-Pidou 3, 1007 Lausanne
9. Chemin Jaques-Larguier-des-Bancels 2, 1004 Lausanne
10. Avenue Collonges 2, 1004 Lausanne
11. Avenue de Beaulieu 19, 1004 Lausanne
12. Sauvabelin drugstore, Route Aloys-Fauquez 87, 1018 Lausanne
13. Route Aloys-Fauquez 54, 1018 Lausanne
14. Chemin François-de-Lucinge 2, 1006 Lausanne
15. Chemin de Montelly 41, 1007 Lausanne
16. Chailly Police Station, Avenue de chailly 5, 1012 Lausanne
17. Avenue de Morges 153, 1004 Lausanne
18. Chemin de Maillefer 117, 1018 Lausanne.
19. Rue des Terreaux 22, 1003 Lausanne
20. Rue du Simplon 25, 1003 Lausanne
21. Bus Station “Faverges”, 1003 Lausanne
22. Rue de la Gare 11a, 1110 Morges
23. Grand Rue 89, 1844 Villeneuve
24. Chemin de Muraz 13, 1814 La Tour-de-Peilz
25. Police Station, Rue du lac 118, 1815 Clarens
26. Rue de l’Eglise Catholique 8, 1820 Montreux
27. Rue de l’Union 15, 1800 Vevey
28. Avenue de Corsier 23, 1800 Vevey
29. Rue des Chenevières 11, 1800 Vevey
30. Rue de Lausanne 6, 1800 Vevey
31. Avenue des Crosets 35, 1800 Vevey
32. Migros market, Rue des Terreaux 22, 1350 Orbe
33. Rue Jules-Gachet 5, 1260 Nyon
34. Chemin d'Eysins 32, 1260 Nyon
35. Rue des Moulins 97, 1400 Yverdon-les-Bains
36. Chemin de Riettaz 3, 1030 Bussigny
37. Rue du Centre 7, 1030 Bussigny
38. Rue de la Mèbre, 1020 Renens, near to the MacDonalds restaurant car park
39. Rue de l'industrie 2, 1020 Renens
40. Chemin des Noutes 15, 1023 Cressier
