# Supplementary material for: How to improve automated external defibrillator placement for out-of-hospital cardiac arrests: A case study
Source: PLoS One. 2021 May 20;16(5):e0250591. doi: 10.1371/journal.pone.0250591 (PMC8136701; doi:10.1371/journal.pone.0250591)
Supplement: S2 File — (DOCX) [file pone.0250591.s002.docx]

# **S2 File. Suggested public AEDs to be removed**

The devices are presented below as follows: identification number, location, address

1. 76141, Pharmacy Place-neuve Benu, Place de la Gare 1, 1009 Pully
2. 76139, Pharmacy Amavita Pulliérane, Rue de la Poste 26, 1009 Pully
3. 76136, SICPA, Route de Renens 24, 1008 Prilly
4. 121434, Swissgrid AG, Route des Flumeaux 41, 1008 Prilly
5. 102108, Salle des Remparts, Place des Anciens-Fossés 7, 1814 La Tour-de-Peilz
6. 76184, Pharmacy Centrale Emery, Rue du lac 27, 1400 Yverdon-les-Bains
7. 76185, Pharmacy Benu du Valentin, Rue Valentin 18, 1400 Yverdon-les-Bains
8. 99351, Unithèque, Université Lausanne, Dorigny, 1015 Lausanne
9. 76023, Service des automobiles et de la navigation (halle technique), Avenue du Grey 110, 1018 Lausanne
10. 76028, Centre Santé au Travail, Route de Berne 52, 1010 Lausanne
11. 106950, Carrosserie Paudex, Route du Lac 1, 1094 Paudex
12. 79372, Contrôle des habitants, Rue du château 1, 1095 Lutry
13. 4672, Andritz Hydro AG, Rue des deux Gares 6, 1800 Vevey
14. X14I708365, Complexe sportif des Mousquetaires, Place des anciens Fossés 13, 1814 La Tour de Peilz
15. 76178, Centre Saint Roch, Route des pêcheurs 8a, 1400 Yverdon-les-bains
16. 76251, Etablissement Cantonal d’Assurance, Av. Général Guisan 54, 1009 Pully
17. A08C 05434, Centre Courrier de la Poste (sous-sol), ZI le Marais, 1300 Eclépens
