# Supplementary material for: How to improve automated external defibrillator placement for out-of-hospital cardiac arrests: A case study
Source: PLoS One. 2021 May 20;16(5):e0250591. doi: 10.1371/journal.pone.0250591 (PMC8136701; doi:10.1371/journal.pone.0250591)
Supplement: S3 File — (DOCX) [file pone.0250591.s003.docx]

**S3 File: Comparison of published AED coverage rates with those obtained in the present study**

| **Author** | **Territory and year** | **Coverage rate** | | | | | **Median straight distance between OHCAs and the nearest AED (meters)** | | | | |
| --- | --- | --- | --- | --- | --- | --- | --- | --- | --- | --- | --- |
|  | | Urban | Rural | Public | Home | All | Urban | Rural | Public | Home | All |
| Tierney et al. [1] | Ticino (Swiss state)  (2005-2015) | 31% | 18% | - | - | 23% | 168 | 269 | - | - | 224 |
| Neves Briard et al. [2] | Montreal area (2014-2015) | - | - | - | - | 2% | - | - | - | - | - |
| Sondergaard et al. [3] | Denmark  (2008-2013)^1^ | - | - | - | 2.0% | 4.6% | - | - | - | - | 800 |
| Fredman et al. [4] | Sweden  (2014) | - | - | - | - | 6.6% | - | - | - | - | - |
| Chan et al. [5] | Toronto  (2005-2010)* | - | - | 23% | - | - | - | - | - | - | - |
| Sun et al. [6] | Toronto  (2006-2014)* | - | - | 18.5% | - | - | - | - | - | - | - |
| Agerskov et al. [7] | Copenhagen  (2011-2013) | - | - | - | - | 23.4% | - | - | - | - | - |
| Hansen et al. [8] | Copenhagen  (1994-2011)* | - | - | 28.8% | - | - | - | - | - | - | - |
| Sun et al. [9] | Copenhagen  (2007-2016)* | - | - | 22.0% | - | - | - | - | - | - | - |
| Fan et al. [10] | Hong Kong  (2012-2013) | - | - | - | - | 30% | - | - | - | - | - |
| Ho et al. [11] | Hong Kong  (2010-2013) | - | - | 25.2% | 25.2% | 25.2% | - | - |  | 167.3 | - |
| Deakin et al. [12] | England (South Central)  (2014-2016) | - | - | - | - | - | - | - | - | - | 1022 |
| Srinavasan et al. [13] | Pittsburgh  (2009-2014) | - | - | - | - | - | - | - | - | - | 515 |
| **Present study** | **Vaud (Swiss state)**  **(2014-2018)** | **10.3%** | **2.9%** | **19.1%** | **4.5%** | **7.5%** | **319.7** | **1874.7** | **303.1** | **429.4** | **410.9** |
| **Present study** | **Lausanne city**  **(2014-2018)** | **13.7%** | **-** | **30.3%** | **7.5%** | **13.7%** | **246.0** | **-** | **172.0** | **268.8** | **246.0** |

^1^ This study only considers AEDs available at the time of the OHCA; therefore, the results could be underestimated compared to other studies that consider AEDs to be available all the time

* These studies have excluded at-home OHCAs

**References**

[1] Tierney NJ, Reinhold HJ, Mira A, Weiser M, Burkart R, Benvenuti C, et al. Novel relocation methods for automatic external defibrillator improve out-of-hospital cardiac arrest coverage under limited resources. Resuscitation. 2018;125: 83–9.

[2] Neves Briard J, de Montigny L, Ross D, de Champlain F, Segal E. Is distance to the nearest registered public automated defibrillator associated with the probability of bystander shock for victims of out-of-hospital cardiac arrest? Prehosp Disaster Med. 2018;33: 153–9.

[3] Sondergaard KB, Hansen SM, Pallisgaard JL, Gerds TA, Wissenberg M, Karlsson L, et al. Out-of-hospital cardiac arrest: probability of bystander defibrillation relative to distance to nearest automated external defibrillator. Resuscitation. 2018;124: 138–44.

[4] Fredman D, Svensson L, Ban Y, Jonsson M, Hollenberg J, Nordberg P, et al. Expanding the first link in the chain of survival – experiences from dispatcher referral of callers to AED locations. Resuscitation. 2016;107: 129–34.

[5] Chan TCY, Li H, Lebovic G, Tang SK, Chan JYT, Cheng HCK, et al. Identifying locations for public access defibrillators using mathematical optimization. Circulation. 2013;127: 1801–9.

[6] Sun CLF, Demirtas D, Brooks SC, Morrison LJ, Chan TCY. Overcoming spatial and temporal barriers to public access defibrillators via optimization. J Am Coll Cardiol. 2016;68: 836–45.

[7] Agerskov M, Nielsen AM, Hansen CM, Hansen MB, Lippert FK, Wissenberg M, et al. Public access defibrillation: great benefit and potential but infrequently used. Resuscitation. 2015;96: 53–8.

[8] Hansen CM, Wissenberg M, Weeke P, Ruwald MH, Lamberts M, Lippert FK, et al. Automated external defibrillators inaccessible to more than half of nearby cardiac arrests in public locations during evening, nighttime, and weekends. Circulation. 2013;128: 2224–31.

[9] Sun CLF, Karlsson L, Torp-Pedersen C, Morrison LJ, Brooks SC, Folke F, et al. In silico trial of optimized versus actual public defibrillator locations. J Am Coll Cardiol. 2019;74: 1557–67.

[10] Fan M, Fan K, Leung L. Walking route–based calculation is recommended for optimizing deployment of publicly accessible defibrillators in urban cities. J Am Heart Assoc. 2020;9: e014398

[11] Ho C, Lui C, Tsui K, Kam C. Investigation of availability and accessibility of community automated external defibrillators in a territory in Hong Kong. Hong Kong Med J. 2014;20: 371–8.

[12] Deakin CD, Anfield S, Hodgetts GA. Underutilisation of public access defibrillation is related to retrieval distance and time-dependent availability. Heart Br Card Soc. 2018;104: 1339–43.

[13] Srinivasan S, Salerno J, Hajari H, Weiss LS, Salcido DD. Modeling a novel hypothetical use of postal collection boxes as automated external defibrillator access points. Resuscitation. 2017;120: 26–30.
